# Back to school: challenges and rewards of engaging young children in scientific research

**DOI:** 10.1136/archdischild-2015-310347

**Published:** 2016-04-26

**Authors:** Janet Stocks, Sooky Lum

**Affiliations:** Respiratory, Critical Care and Anaesthesia section (Portex Unit), UCL Institute of Child Health, London, UK

**Keywords:** children, Health services research, recruitment

The great comedian, W C Fields, is credited with the line, “Never work with children or animals”, but as those of us who have spent a lifetime in the field know, nothing could be further from the truth when engaging in medical research that relates to children. Nevertheless, embarking or participating in paediatric research can be a daunting prospect for those unfamiliar to engaging children in such activities. The aim of this article is to share some of our recent experience in this field in order to encourage others to engage in this hugely worthwhile field.

As is now well recognised, children are not small adults but have an additional, unique set of interests and needs. Paediatricians can play a pivotal role in facilitating paediatric research, since many parents will seek their doctor's advice regarding potential participation in a research project. Unfortunately doctors' reluctance about their patients' participation in research, whether on the grounds of lack of equipoise or the additional workload that this may entail, remains one of the major barriers to research.^1^ Public trust in paediatric research is also crucial. In order for much needed research to proceed, the public needs to know that standards are in place and are adhered to, to protect the interests of children. Involvement of service users including children in decision-making, in both clinical and research settings, has become a central feature of many health and research funding policies in the past 15 years. Although beyond the scope of the current article, the urgent need to address the current lack of evidence relating to children's collaboration and involvement *with* research has been summarised recently.^2^ What is however beyond doubt is that research involving children is important for the benefit of all children, provided it is carried out in an ethical manner^1^ ^3–5^ and is highly rewarding for those undertaking it and can provide valuable training that may improve the quality of clinical practice (http://www.phc.ox.ac.uk/research/childrens-health). The Nuffield Council on Bioethics have challenged the idea that clinical research is something from which children need to be protected and essentially excluded from and have recently published recommendations with which to conduct relevant clinical research in order to protect children and young people *through* research.^6^

Given the many challenges to be faced when undertaking research in children, it is essential to ascertain before starting that it is worthwhile in terms of being of benefit to children, not duplicating earlier work, being well designed and conducted, including a statistically appropriate number of subjects, being feasible in the relevant age group and leading to definitive results that can be properly reported, all of which is required before submitting any proposal to an ethics committee for approval. This requires a thorough search of the literature, discussion with experts in the field and engagement of a suitably qualified statistician during the early stages of planning the study design and appropriate communication with all relevant members of the wider community, the nature of which will vary according to where the research will be undertaken.

The specific challenges and rewards associated with research in children will depend on specific circumstances, including clinical status (ranging from completely healthy to those who are critically ill), age and the precise research setting (eg, home, school, laboratory, medical centre or hospital-based venue). While it may be relatively easy to recruit children with specific diseases while in hospital as they and their families have most to gain from targeted research, this potential advantage may be counteracted by practical problems, such as parental anxiety, the child being too ill to study or the need for an immediate intervention which precludes the ability to obtain prospective parental consent.^7^ In instances such as encountered in emergency medicine, use of deferred consent may be preferable. Under such circumstances, the Consent Methods in Children's Emergency Medicine and Urgent Care Trials guidance will help practitioners to conduct research without prior consent in a way that is ethically appropriate and addresses the needs of families.^8^ ^9^

Alternatively, the disease under investigation may be so rare that it is difficult to recruit sufficient children to reach definitive conclusions in a timely manner, especially given the rate at which medical and technological advances are currently being made. Some of these issues are currently being addressed by the proposed development of ‘generic consent’ schemes in which all parents would be asked on arrival at major teaching hospitals whether they would be willing for their or their child's clinical information to be made available for research, provided all data were anonymised and used within a strict ethical framework.^10^ Indeed, recent genetic breakthroughs involving the sequencing of 100 000 human genomes in order to identify ‘mystery diseases’ owe their success to the existence of the NHS in Britain, with its potential for storing and sharing data on a massive scale.^11^ However, as discussed below, even with full parental consent, access to routine NHS data is not as straightforward as might be hoped for.

Other approaches to conducting research in children with relatively rare diseases such as cystic fibrosis (CF), where specialised measurements additional to routine investigations may be required, are illustrated by the success of the London CF Collaboration (LCFC) (http://www.ucl.ac.uk/london-cystic-fibrosis). This involved all the CF paediatric specialist hospitals in London working together to investigate the natural history of lung disease in children with CF both before and after the introduction of national newborn screening was introduced in the UK in 2007. Results from this group have demonstrated that lung function can deteriorate soon after birth and before symptoms are present,^12^ that lung function during the preschool years is predictive of that in later school age^13^ and that there has been a significant improvement in lung function in infants with CF since newborn screening was introduced, such that at 1 year of age, any structural changes are generally too mild to detect reliably, precluding routine use of CT scans at this age.^14^ ^15^ The LCFC have also demonstrated the feasibility and enormous value of recruiting contemporaneous healthy controls at all stages of their research programme in order to optimise interpretation of results.^16–18^

## Recruitment of healthy controls

The ability to recruit healthy children into non-interventional medical and scientific research presents its own special challenges. While the children themselves may be eager to participate, parents may be anxious about potential risks or unable to commit time to attend appointments due to ongoing family and work commitments.^19^ ^20^ To improve recruitment, many studies in school-aged children have been designed to allow data collection within the school environment.^21–23^ This approach minimises the burden on parents and reduces the risk of selection bias, either with respect to socioeconomic circumstances or the chance that parents of children with perceived problems may be more likely to consent to participation. As discussed below, our recent experience of engaging a large number of healthy London primary school children into a multidisciplinary, multi-ethnic research study funded by the Wellcome Trust: The Size and Lung function In Children (SLIC) study^24^ illustrates some of the challenges and rewards of undertaking this type of non-interventional research in the community as well as the importance of detailed planning and good communication at all levels.

### Importance of a pilot study

Definitive funding for large epidemiological studies such as the SLIC study is unlikely to be forthcoming unless the proposal has been shown to be feasible. As such, an initial pilot study to trial all aspects of the study from recruitment, consent and methodological issues to data collection and management can be invaluable and many charitable organisations now provide limited ‘pump-priming’ or seed-funding to undertake such work.^25^ ^26^ Such a pilot study can provide the opportunity to ensure that suitable equipment has been selected for field conditions, in terms of portability, robustness and avoidance of calibration issues such as may occur in unduly hot or cold environments and that any questionnaires are ‘fit for purpose’. In addition to providing the opportunity to develop and implement standardised protocols, thorough training of researchers and ongoing quality assurance checks throughout the study, it will highlight the importance of factors such as optimal duration, timing and location of assessments to ensure that the study protocol causes minimal disruption within the school timetable. An appropriate pilot study will also facilitate a realistic assessment of the number of children needing to be recruited to achieve the required sample size, after allowing for issues such as consent, failure rates and exclusions on health grounds.^25^

### Recruitment of schools

To minimise bias when recruiting for paediatric studies within a multi-ethnic population, schools with a high ethnic mix need to be identified and sampled by education performance within boroughs to ensure a wide range of socioeconomic circumstances prior to initial contact with suitable head teachers. In addition to sending both a brief summary and full details of the study (suitably worded to avoid all jargon) to those showing potential interest, a personal visit by the lead investigators to each school is mandatory, both to check physical location and layout and to ensure the best possible communication with the head teacher the school administrators, caretaker and ideally some of the teaching staff.

### Public engagement

Following agreement by the school to participate, the study needs to be publicised as widely as possible by using school newsletters and leaflets, by local media and by inviting parents to attend presentations in the evenings or end of school day. Head teachers, teaching staff and parent representatives should be consulted on how best to raise awareness and explain the purpose and nature of the study to children and their families. For research studies focused on issues from non-English-speaking communities, it may be helpful to get the involvement of community leaders or well-known local personalities to support and promote the research, and recruitment may be also extended to local temples or mosques. Publicity for the study may also be achieved through local media via local radio or weekly newspapers in their specific languages. In such studies, representatives from the schools and local community should form part of the Project Steering Committee, whose main aim is to monitor and supervise the progress of the study towards its interim and overall objectives.

### Recruitment of children

Interactive science workshops,^24^ adapted according to the specific research question, subject's age group and if possible the school curriculum, can greatly increase children's enthusiasm to participate, the latter being key to obtaining parental consent. Given that most children have an inherent competitive streak, an interschool or interclass competition may also help boost study recruitment, as will a personal recognition for each child's participation using appropriate incentives such as a medal or certificate. It should be noted that in Europe (although not in the USA), incentive payments for paediatric research are illegal.^1^ ^27^ To avoid the potential personal distress to a child that exclusion on health grounds might cause when recruiting within a school setting, as well as any potential breach of medical confidentiality, an ‘inclusive’ approach is advisable, whereby no willing child is excluded from testing, their data simply being excluded from subsequent definitive analyses if specified inclusion criteria have not been met.^25^ ^28^ One advantage of this approach is the ability to objectively assess the extent to which inclusion and exclusion criteria actually impact on population-based studies.^28^

To encourage enthusiasm and retention of schools/subjects during longitudinal studies, presentation of study updates to staff, parents and at school assemblies, together with the availability of a study website (eg, http://www.ucl.ac.uk/slic),^24^ will enable all interested parties to follow the study progress and retain a sense of ‘belonging’. At the end of the study it is also important to request feedback from children, parents and teachers to inform future studies.^24^

### Challenges of field studies

When undertaking research in inner city schools, location of adequate space in which to conduct assessments may impact on which schools can be selected for recruitment. For example, even if there is space for a mobile laboratory within the playground/school grounds, location of a school next to a busy road with heavy traffic may compromise equipment function. Whatever the circumstances, it will always be essential to work closely with school staff to identify the most appropriate location and ensure that the children's safety and access is not compromised.

While placing far less burden on parents, since their attendance is generally not required during data collection, there are methodological limitations associated with school-based research. Children will need to take relevant paperwork such as information sheets, questionnaires and consent forms home (and return them!) and the research team will be dependent on parents completing the study questionnaire accurately if essential background information such as ethnicity, birth and medical history of the child and family is to be recorded. Potentially sensitive and complex issues such as categorisation of socio-economic circumstances and ethnicity may not be reported reliably. Furthermore, accurate categorisation of pubertal status is difficult especially in primary school children as self-report has been shown to be unreliable.^29^

With increasing computerisation of health records, checking basic details such as birth weight, gestational age and prior/current medication and diagnosis should be relatively straightforward via the child's general practitioner (GP/family doctor). However, while parental consent to access GP records was received from 95% of children participating in the SLIC study, only 40% of GP practices across six NHS Trust across London agreed to participate, despite the offer of monetary incentives and the availability of a member of the research team to extract the necessary information. In addition, the response rate from GPs was especially low for children from more deprived areas, thereby risking collection bias and information less likely to be available had the child been born outside the UK. Fortunately, parental report of birth data was found to be an appropriate alternative to health records in this study.^30^

## Conclusion

Despite the ethical and practical challenges of involving children in research, it is imperative that the paediatric medical community advocate for more research both for children and with children and to ensure that such research is conducted to the highest possible standards. Given the importance of primary care in shaping the treatment that children receive, it is of particular importance to engage GPs and parents when discussing priorities for future research in this age group. There is little doubt that undertaking research with young children can be exhausting and presents its own specific challenges, but these are more than compensated by the results that can be achieved and by the sheer enthusiasm with which young people engage in such activities.
